# Guideline-Directed Medical Therapy Intensity, Ventricular Remodeling, and Clinical Outcomes After Acute Myocardial Infarction: A Single-Center Real-World Retrospective Cohort Study

**DOI:** 10.3390/biomedicines14051067

**Published:** 2026-05-08

**Authors:** Teodora Mateoc-Sîrb, Ioana-Maria Suciu, Dan Gaiță, Andor Minodora, Roxana Popescu, Tania Vlad, Călin Muntean, Daliborca-Cristina Vlad

**Affiliations:** 1 Doctoral School, Faculty of Medicine, “Victor Babeș” University of Medicine and Pharmacy, 2nd Eftimie Murgu Square, 300041 Timisoara, Romania; teodora.mateoc@umft.ro (T.M.-S.); vlad.daliborca@umft.ro (D.-C.V.); 2 Institute of Cardiovascular Diseases Timișoara, No. 13A Gheorghe Adam Street, 300310 Timisoara, Romania; dgaita@cardiologie.ro; 3 Multidisciplinary Heart Research Center, “Victor Babeș” University of Medicine and Pharmacy, 300041 Timisoara, Romania; andor.minodora@umft.ro; 4 Medical Semiology II Discipline, Internal Medicine Department, “Victor Babeș” University of Medicine and Pharmacy, 2nd Eftimie Murgu Square, 300041 Timisoara, Romania; 5 Department of Biochemistry and Pharmacology, Faculty of Medicine, “Victor Babeș” University of Medicine and Pharmacy, 2nd Eftimie Murgu Square, 300041 Timisoara, Romania; popescu.roxana@umft.ro (R.P.); tania.vlad@umft.ro (T.V.); 6 Department III-Functional Sciences, Medical Informatics and Biostatistics, “Victor Babeș” University of Medicine and Pharmacy, 2nd Eftimie Murgu Square, 300041 Timisoara, Romania; cmuntean@umft.ro

**Keywords:** guideline-directed medical therapy, heart failure, acute myocardial infarction, ventricular remodeling, implementation gap, SGLT2 inhibitors

## Abstract

**Background:** Guideline-directed medical therapy (GDMT) is recommended after acute myocardial infarction (AMI), particularly in patients with left ventricular systolic dysfunction, yet real-world implementation remains suboptimal. Whether greater early GDMT intensity is associated with post-infarction ventricular remodeling has not been fully established. We aimed to quantify the guideline-to-practice gap and evaluate the association between GDMT intensity, cardiac remodeling, and clinical outcomes after AMI. **Methods:** In this single-center retrospective cohort study, 186 consecutive patients hospitalized for AMI who underwent successful percutaneous coronary intervention and had baseline plus follow-up transthoracic echocardiography were included. GDMT intensity was defined as the number of prescribed foundational therapy classes at discharge (renin–angiotensin system inhibitors, beta-blockers, mineralocorticoid receptor antagonists, and sodium–glucose cotransporter 2 inhibitors; range 0–4). The primary endpoint was change in left ventricular end-diastolic diameter (ΔLVEDD). Secondary endpoints included changes in left ventricular ejection fraction, left ventricular end-diastolic volume, left ventricular mass, and heart failure rehospitalization. Multivariable models adjusted for relevant clinical covariates were applied. **Results:** Only 18.8% of the overall cohort and 26.2% of patients with baseline left ventricular ejection fraction ≤ 40% received all four GDMT pillars. A graded association was observed between higher GDMT intensity and more favorable remodeling. In adjusted analyses, each additional GDMT pillar was associated with a greater reduction in LVEDD (β = 0.120 cm, *p* = 0.004). In the prespecified reduced-ejection-fraction subgroup, the association was stronger (β = 0.204 cm, *p* < 0.001). Higher GDMT intensity was also associated with lower odds of heart failure rehospitalization (odds ratio 0.384, 95% CI 0.195–0.754; *p =* 0.006). **Conclusions:** In this real-world post-AMI cohort, broader implementation of foundational GDMT at discharge was associated with more favorable early ventricular reverse remodeling and lower odds of heart failure rehospitalization. These findings highlight a persistent implementation gap and support prospective studies evaluating rapid comprehensive GDMT initiation after AMI.

## 1. Introduction

Heart failure (HF) is a major driver of morbidity and mortality after acute myocardial infarction (AMI), with guideline-directed medical therapy (GDMT) serving as the cornerstone of management [1]. The 2021 European Society of Cardiology (ESC) Guidelines for heart failure radically shifted the treatment paradigm by recommending the rapid, simultaneous initiation of four foundational therapeutic pillars for patients with HF and reduced ejection fraction (HFrEF): renin–angiotensin system inhibitors (RAASi), beta-blockers (BB), mineralocorticoid receptor antagonists (MRA), and sodium-glucose cotransporter 2 inhibitors (SGLT2i) [2]. This strategy aims to achieve early and comprehensive neurohormonal and metabolic blockade, which has been associated with more favorable clinical outcomes.

The urgency of this approach was validated by the STRONG-HF trial [3], which showed that intensive, rapid up-titration of oral HF therapies before hospital discharge significantly reduced the 180-day risk of HF rehospitalization or all-cause death. Consequently, the 2023 ESC Focused Update elevated this intensive strategy to a Class I recommendation and broadened the Class I indication for SGLT2i to include patients across the full LVEF spectrum (HFmrEF and HFpEF) [4], based on landmark trials like EMPEROR-Preserved [5,6] and DELIVER [7]. Beyond the four foundational GDMT pillars, recent therapeutic advances continue to expand the HFrEF treatment landscape. In an individual participant data analysis of the VICTORIA and VICTOR trials, vericiguat was associated with clinical benefit across a broad risk spectrum of patients with HFrEF, reinforcing the value of comprehensive pathway modulation in contemporary heart failure care [8].

Despite this compelling evidence and unequivocal guideline consensus, a profound gap persists between these recommendations and real-world clinical practice. International registries consistently demonstrate significant underutilization of GDMT [9], a phenomenon termed therapeutic inertia, which is particularly pronounced in the post-AMI setting. While the clinical benefits of GDMT are well-documented, the direct association between the intensity of in-hospital therapy (i.e., the number of pillars initiated) and early markers of structural cardiac remodeling remains insufficiently characterized [10]. Understanding these early structural correlations is critical, as they provide a mechanistic rationale for overcoming implementation barriers and may serve as surrogate endpoints for long-term clinical benefit.

Because post-infarction ventricular remodeling may develop across the left ventricular ejection fraction (LVEF) spectrum, we included a consecutive post-AMI cohort with serial echocardiographic assessment in order to examine structural remodeling in real-world practice. However, because the strongest guideline support for simultaneous initiation of all four GDMT pillars remains in patients with HFrEF, we prespecified a subgroup analysis in patients with baseline LVEF ≤ 40%, while analyses in patients with LVEF > 40% were considered exploratory and hypothesis-generating.

This study was designed to address this evidence–practice gap. We aimed to (1) quantify the real-world implementation rates of the four GDMT pillars in a contemporary post-AMI cohort, benchmarking our findings against ESC guideline targets; and (2) investigate the association between GDMT intensity and a comprehensive panel of subsequent echocardiographic remodeling parameters and clinical outcomes. We hypothesized that greater GDMT intensity would be associated with more favorable reverse remodeling, while exploratory analyses would evaluate whether higher treatment intensity was also linked to lower odds of heart failure rehospitalization.

Accordingly, the present study should be interpreted primarily as a real-world investigation of post-infarction ventricular remodeling in relation to treatment intensity, with prespecified HFrEF subgroup analyses reflecting the population with the strongest guideline support for four-pillar GDMT.

## 2. Methods

### 2.1. Study Design and Population

This was a single-center retrospective observational cohort study conducted at the Institute of Cardiovascular Disease in Timișoara, Western Romania. The protocol was elaborated in full compliance with the STROBE (Strengthening the Reporting of Observational Studies in Epidemiology) recommendations [11], ensuring transparent and comprehensive reporting regarding study design, participant selection, variable definitions, and statistical analysis methods. Data were collected for the period between January 2023 and October 2024. Owing to the retrospective design, the requirement for informed patient consent was waived. Ethical clearance was granted by the institutional review board (protocol code 5301; approval date: 9 July 2024), and all study procedures adhered to the ethical standards outlined in the Declaration of Helsinki.

### 2.2. Data Collection and Definitions

#### 2.2.1. Data Collection

Data were retrospectively collected from the hospital’s electronic health records using a standardized data collection form. Baseline data included patient demographics, cardiovascular risk factors (hypertension, diabetes mellitus, dyslipidemia, smoking status), clinical presentation, laboratory values, and comprehensive echocardiographic measurements performed during the index hospitalization. Follow-up data, including clinical events and repeat echocardiography, were collected from the first subsequent cardiology evaluation. As an exploratory retrospective cohort study, no prospective sample size calculation was performed; all eligible consecutive patients during the study period were included.

#### 2.2.2. GDMT Exposure Definition

The main exposure variable was the intensity of GDMT, defined as the number of HF therapy pillars (RAASi, BB, MRA, SGLT2i) initiated during the index hospitalization and prescribed at discharge. GDMT intensity was quantified as an ordinal variable ranging from 0 to 4. Medication exposure was operationalized according to therapies prescribed at hospital discharge. Due to the retrospective structure of the dataset, distinction between de novo initiation during index hospitalization and continuation of pre-existing outpatient therapy was not consistently available for all drug classes. Accordingly, GDMT intensity should be interpreted pragmatically as discharge treatment intensity rather than confirmed de novo in-hospital initiation.

This exposure variable reflects therapeutic breadth (i.e., the number of foundational classes initiated during index hospitalization) rather than pharmacologic dose intensity. Information on initial dose, post-discharge up-titration, and achievement of guideline-recommended target doses was not consistently available in the retrospective dataset. To reduce overfitting risk, clinically selected covariates were limited a priori.

#### 2.2.3. Study Population and Eligibility Criteria

The study population included consecutive adult patients admitted to the coronary care unit with a confirmed diagnosis of acute coronary syndrome (ACS). Patients were eligible if they met the following criteria: (1) verified diagnosis of ACS, classified as either ST-segment elevation myocardial infarction (STEMI) or non-ST-segment elevation acute coronary syndrome (NSTE-ACS); (2) successful percutaneous coronary intervention (PCI) performed during the index admission; and (3) availability of a comprehensive transthoracic echocardiographic (TTE) assessment at baseline, along with at least one follow-up TTE conducted between 3 and 34 months after hospital discharge.

We intentionally did not restrict the primary cohort to HFrEF because the biological process of post-infarction left ventricular remodeling is not confined to patients with LVEF ≤ 40%, and several GDMT components may also be prescribed after AMI or across broader HF phenotypes according to the clinical context. Nevertheless, the subgroup with baseline LVEF ≤ 40% was predefined as the most guideline-concordant population for evaluating full four-pillar GDMT intensity.

Out of 382 individuals initially screened, 186 fulfilled all inclusion and exclusion criteria and were retained for the final analysis. The cohort was subsequently divided into two subgroups: patients with reduced ejection fraction (LVEF ≤ 40%) (*n* = 107) and those with moderately reduced or preserved ejection fraction (LVEF > 40%) (*n* = 79).

Patients were excluded in the presence of incomplete echocardiographic (*n* = 89) or laboratory data (*n* = 32), advanced renal failure (estimated glomerular filtration rate < 20 mL/min/1.73 m^2^) or requirement for dialysis (*n* = 8), active cancer (*n* = 9), sepsis (*n* = 6), or rehospitalization for unstable angina or acute myocardial infarction (*n* = 52).

#### 2.2.4. Echocardiographic Assessment

Transthoracic echocardiography was conducted using a Vivid S5 system (GE Healthcare, Chicago, IL, USA) by different operators and following recommended guidelines [12]. The following parameters were assessed: left ventricular ejection fraction (LVEF), determined by the biplane Simpson method; left ventricular mass (LVM), calculated using the linear method according to the Devereux formula; indexed left ventricular mass (LVMi), obtained by normalizing LVM to body surface area (BSA); relative wall thickness (RWT), computed as (2 × posterior wall thickness [PW]) divided by left ventricular end-diastolic diameter (LVEDD); and longitudinal change (Δ), defined as the difference between the baseline and follow-up values (baseline minus follow-up).

All remodeling parameters were expressed as the difference between baseline and follow-up measurements (Δ = baseline − follow-up). For structural parameters (LVEDD, LVEDV, and LVM), positive Δ values indicate favorable reverse remodeling, reflecting a reduction in chamber dimensions or myocardial mass. In contrast, for LVEF, negative Δ values indicate functional improvement, corresponding to an increase in ejection fraction at follow-up.

### 2.3. Endpoints

**Figure 1:**
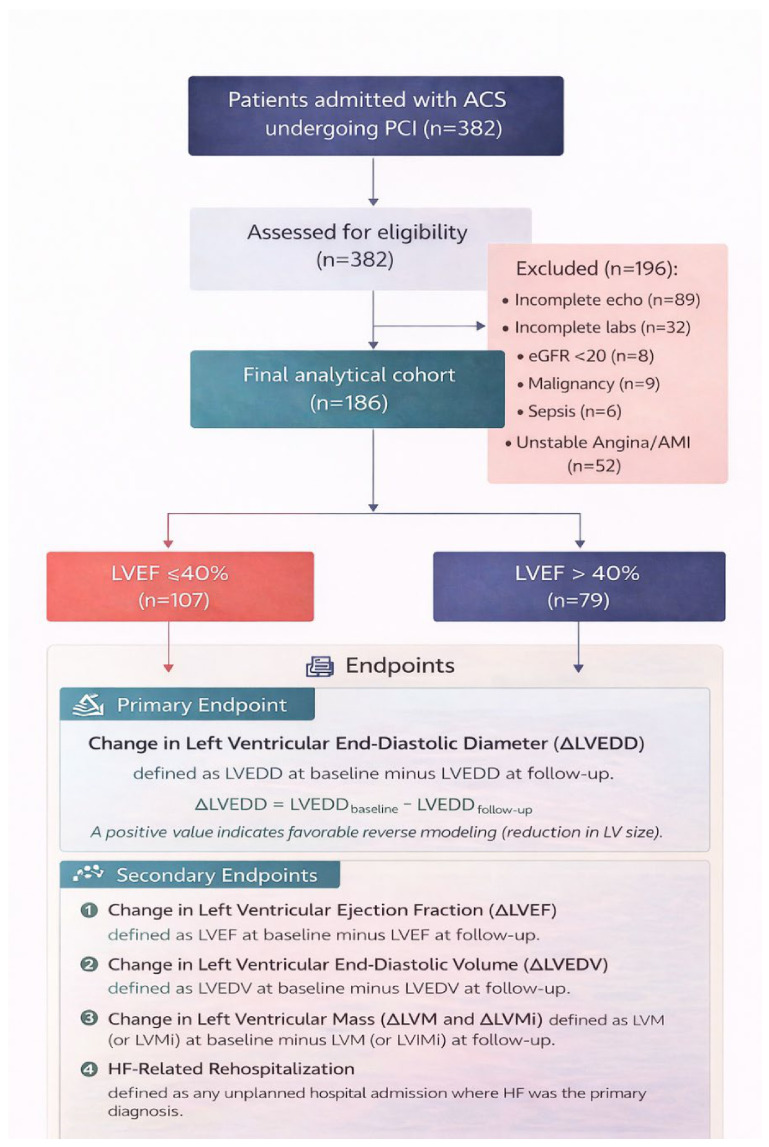
Study flowchart showing patient screening, exclusion criteria, final cohort selection, and subgroup allocation according to baseline left ventricular ejection fraction (LVEF ≤40% vs. >40%).

The primary endpoint was the change in left ventricular end-diastolic diameter (ΔLVEDD) between the baseline and follow-up echocardiograms, defined as LVEDD at baseline minus LVEDD at follow-up (Figure 1). A positive value indicates favorable reverse remodeling (a reduction in LV size).

ΔLVEDD was selected a priori as the primary remodeling endpoint because left ventricular end-diastolic diameter is a simple, reproducible, routinely available echocardiographic marker of post-infarction ventricular dilation and adverse remodeling. Progressive LV enlargement reflects maladaptive structural remodeling and has established prognostic relevance after myocardial infarction. In contrast to LVEF, LVEDD may be less influenced by short-term loading conditions and therefore may provide a more stable early structural measure in retrospective real-world datasets.

Secondary endpoints included

1. Change in left ventricular ejection fraction (ΔLVEF): Defined as LVEF at baseline minus LVEF at follow-up.
2. Change in left ventricular end-diastolic volume (ΔLVEDV): Defined as LVEDV at baseline minus LVEDV at follow-up.
3. Change in left ventricular mass (ΔLVM and ΔLVMi): Defined as baseline LVM minus follow-up LVM, and baseline LVM indexed to body surface area (LVMi) minus follow-up LVMi.
4. Heart failure-related rehospitalization: Defined as any unplanned hospital admission for which HF was the primary diagnosis.

A prespecified subgroup analysis was performed in patients with HFrEF (baseline LVEF ≤ 40%), where the indication for all four GDMT pillars is most strongly established.

### 2.4. Statistical Analysis

Statistical analysis was performed using Python (version 3.11) with the pandas (version 3.0.2), scipy (version 1.17.1), statsmodels (version 0.14.6), and lifelines libraries (version 0.30.3). Continuous variables were expressed as mean ± standard deviation (SD) or median [interquartile range (IQR) and compared using Student’s *t*-test, Mann–Whitney U test, or Kruskal–Wallis test, as appropriate. Categorical variables were presented as counts and percentages and compared using the Chi-square or Fisher’s exact test. The association between ordinal GDMT intensity and continuous remodeling parameters was assessed using Spearman’s rank correlation.

Multivariable linear regression models were constructed to determine the independent association between GDMT pillar count and echocardiographic remodeling parameters (ΔLVEDD, ΔLVEF, ΔLVM), adjusting for clinically relevant confounders including age, sex, ACS type, diabetes, multivessel disease, baseline echocardiographic values, and time to follow-up. A multivariable logistic regression model was used to assess the independent association between GDMT intensity and the odds of HF-related rehospitalization. A two-sided *p*-value < 0.05 was considered statistically significant.

Additional sensitivity analyses included further adjustment for baseline clinical severity markers (Killip class, systolic blood pressure, heart rate, and serum creatinine), stratification by follow-up duration (<6 vs. ≥6 months), and Kaplan–Meier estimation of HF-related rehospitalization with comparison by log-rank testing.

## 3. Results

### 3.1. Baseline Characteristics

The baseline characteristics of the 186 patients stratified by baseline LVEF are presented in Table 1. Patients with HFrEF (LVEF ≤ 40%) were more likely to be male, present with STEMI, have diabetes, and have longer hospital stays. They also had significantly higher rates of HF-related hospitalization during follow-up (15.9% vs. 3.8%, *p* = 0.0017). Notably, patients with HFrEF had a significantly higher prevalence of single-vessel (monovascular) disease (29.9% vs. 19.0%, *p* = 0.013), which may reflect more localized transmural infarctions leading to severe systolic dysfunction.

### 3.2. Guideline-to-Practice Gap in GDMT Implementation

**Figure 2:**
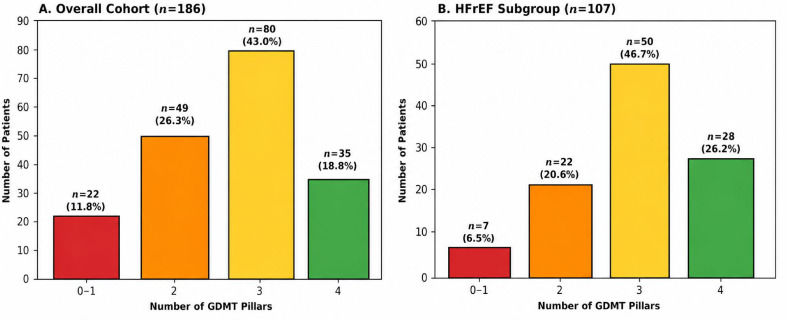
Distribution of guideline-directed medical therapy (GDMT) intensity at hospital discharge. Bar charts show the proportion of patients receiving 0–1, 2, 3, or 4 GDMT pillars in (**A**) the overall post-acute myocardial infarction (AMI) cohort and (**B**) the subgroup with heart failure and reduced ejection fraction (HFrEF; LVEF ≤ 40%).

**Figure 3:**
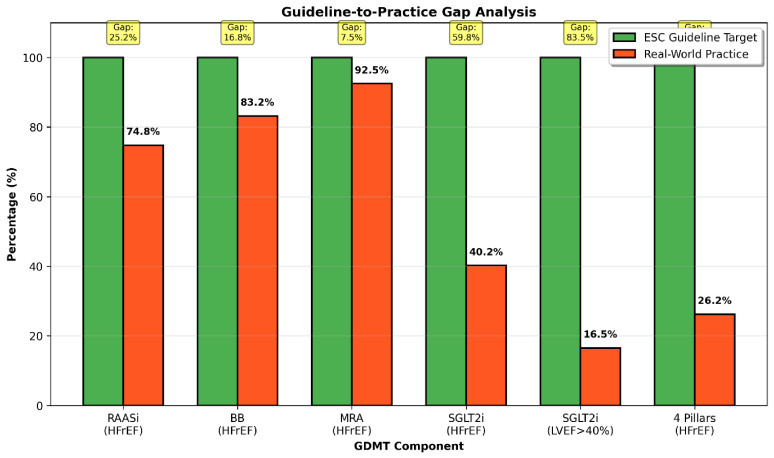
Guideline-to-practice gap in GDMT implementation. Comparison between ideal implementation of Class I guideline-recommended therapies and observed real-world prescription rates at discharge. Percentages above bars indicate observed use; highlighted values represent the estimated implementation gap.

The implementation of GDMT is detailed in Table 2. While MRA were prescribed in 86% of patients, and RAASi and BBs in ~75%, SGLT2i were only initiated in 30.1% of the cohort. In the HFrEF subgroup (Figure 2), where all four pillars are strongly recommended, only 26.2% of patients were discharged on quadruple therapy. The most significant implementation gap was for SGLT2 inhibitors, which were prescribed to only 40.2% of HFrEF patients (Figure 3).

### 3.3. Echocardiographic Remodeling

**Figure 4:**
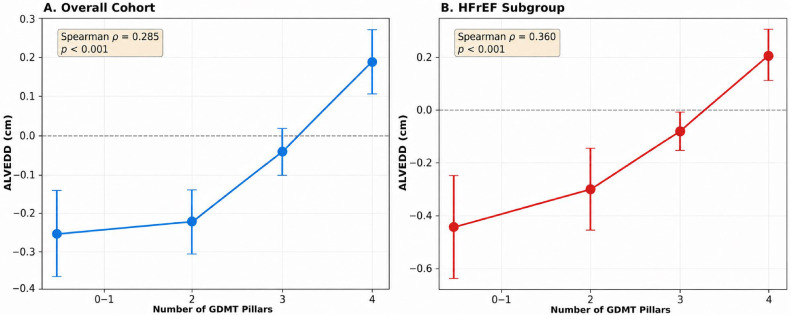
Relationship between GDMT intensity and left ventricular remodeling. Mean change in left ventricular end-diastolic diameter (ΔLVEDD) according to the number of GDMT pillars prescribed at discharge in (**A**) the overall cohort and (**B**) the HFrEF subgroup. Positive ΔLVEDD values indicate favorable reverse remodeling (reduction in LV diameter). Error bars represent the standard error of the mean.

Baseline and follow-up echocardiographic measurements are shown in Table 3. Patients with HFrEF had significantly larger baseline LV dimensions and mass (LVEDD: 4.99 ± 0.62 cm vs. 4.69 ± 0.57 cm, *p* = 0.0009; LVEDV: 125.2 ± 36.9 mL vs. 106.2 ± 26.0 mL, *p* = 0.0001; LVM: 237.4 ± 65.9 g vs. 209.8 ± 58.3 g, *p* = 0.0035). A significant dose–response relationship was observed between GDMT intensity and structural reverse remodeling, with higher pillar counts associated with greater reductions in LV diameter and mass (Figure 4).

### 3.4. Association Between GDMT Intensity and Outcomes in HFrEF

**Figure 5:**
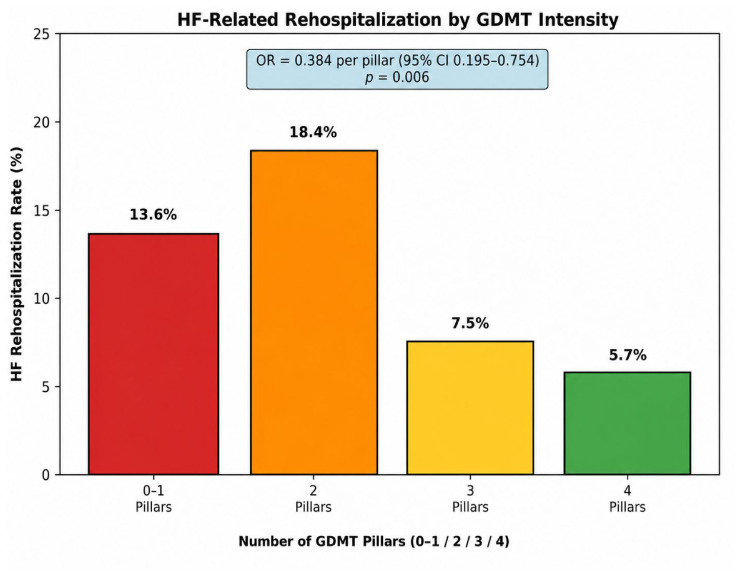
HF rehospitalization according to GDMT intensity. Percentage of patients rehospitalized for heart failure during follow-up, stratified by the number of GDMT pillars prescribed at discharge.

In the prespecified subgroup of 107 patients with HFrEF, the association between GDMT intensity and reverse remodeling was particularly strong (Table 4 and Figure 5). Patients receiving four pillars demonstrated significant reverse remodeling compared to those receiving fewer than four pillars (ΔLVEDD: +0.21 cm vs. −0.17 cm, *p* = 0.0021), indicating a reduction in LV diameter in the fully treated group and mild dilation in the less intensively treated group.

Similar significant or near-significant trends were observed for ΔLVEDV. ΔLVM and ΔLVMi. The observation that the <four pillars group exhibited a negative ΔLVEDD (indicating mild ventricular dilation) alongside a positive ΔLVM (indicating mass reduction) reflects the pathophysiological complexity of post-infarction eccentric remodeling in which ventricular cavity dilation may proceed while myocardial mass decreases due to wall thinning in the infarcted zone and resolution of acute myocardial edema.

**Figure 6:**
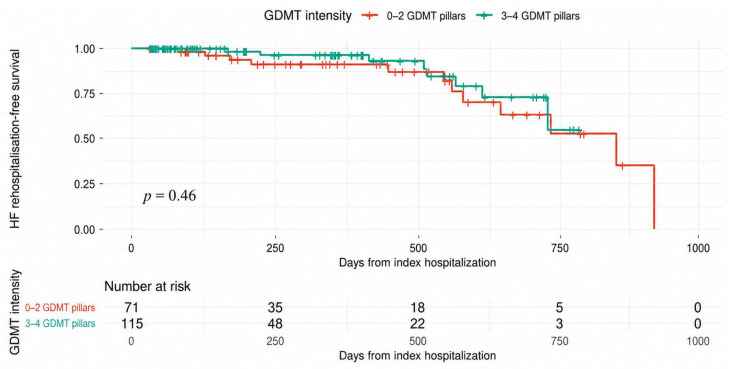
Kaplan–Meier analysis of HF rehospitalization according to GDMT intensity. Patients discharged on higher GDMT intensity (3–4 pillars) were compared with those receiving lower intensity therapy (0–2 pillars). Differences between groups were assessed using the log-rank test (*p* = 0.46). Tick marks indicate censored observations.

Kaplan–Meier analysis of HF-related rehospitalization did not demonstrate a statistically significant difference between patients receiving higher GDMT intensity (3–4 pillars) and those receiving lower intensity therapy (0–2 pillars) (log-rank *p* = 0.46) (Figure 6). Event rates were numerically lower in the higher-intensity group, but this exploratory analysis was limited by the low number of events (Figure 5).

### 3.5. Multivariable Analysis

**Figure 7:**
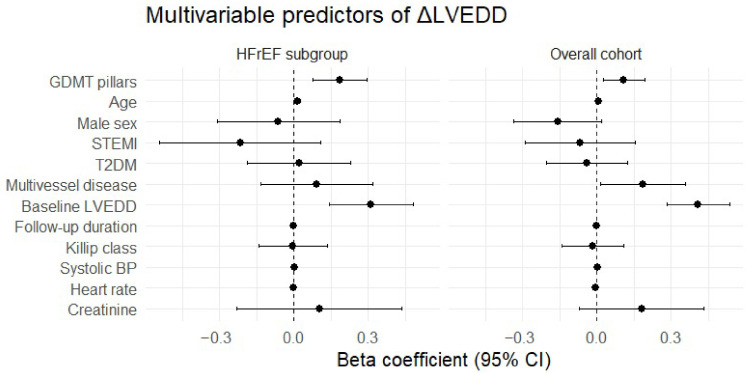
Forest plot of multivariable predictors of favorable remodeling (ΔLVEDD) in the overall cohort and HFrEF subgroup. Points represent beta coefficients, and horizontal lines indicate 95% confidence intervals. Positive beta values indicate greater reduction in left ventricular end-diastolic diameter (more favorable reverse remodeling).

After adjustment for potential confounders (Table 5 and Figure 7), higher GDMT intensity remained independently associated with favorable reverse remodeling, as reflected by greater reduction in LVEDD. In the overall cohort, each additional GDMT pillar was associated with a 0.120 cm larger reduction in LVEDD (*p* = 0.004). This association remained significant after further adjustment for baseline clinical severity markers, including Killip class, systolic blood pressure, heart rate, and serum creatinine (β = 0.109, *p* = 0.010).

In the prespecified HFrEF subgroup, the association was more pronounced. Each additional GDMT pillar was associated with a 0.204 cm larger reduction in LVEDD (*p* < 0.001), remaining significant after additional severity adjustment (β = 0.186, *p* = 0.001).

Higher GDMT intensity was also independently associated with lower odds of HF-related rehospitalization. Each additional GDMT pillar was associated with 62% lower odds of rehospitalization (OR = 0.384, 95% CI 0.195–0.754, *p* = 0.006).

The consistency of findings across progressively adjusted models supports the stability of the observed association between GDMT intensity and post-infarction remodeling.

### 3.6. Sensitivity Analysis According to Follow-Up Duration

**Figure 8:**
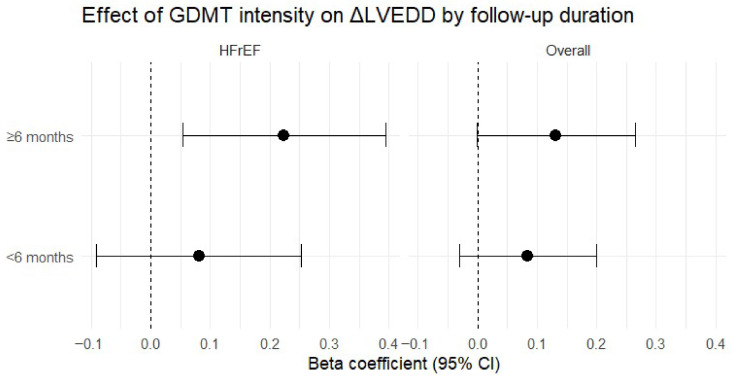
Association between GDMT intensity and ΔLVEDD stratified by follow-up duration.

To address heterogeneity in follow-up duration, sensitivity analyses stratified by follow-up time (<6 months vs. ≥6 months) were performed (Figure 8, Tables S2 and S3). In the overall cohort, the association between GDMT intensity and favorable remodeling was more evident at longer follow-up intervals (β = 0.132, *p* = 0.051) than at shorter follow-up durations (β = 0.084, *p* = 0.150). In the HFrEF subgroup, higher GDMT intensity was significantly associated with greater reverse remodeling among patients with follow-up ≥6 months (β = 0.224, *p* = 0.011), while no significant association was observed at shorter follow-up durations (β = 0.081, *p* = 0.345).

These findings suggest that the association between GDMT intensity and remodeling may be more readily detectable over longer follow-up intervals, particularly in HFrEF.

## 4. Discussion

This study primarily evaluated the relationship between discharge GDMT intensity and early post-infarction ventricular remodeling in a real-world AMI cohort. Greater treatment intensity was associated with more favorable structural remodeling, while exploratory clinical outcome analyses suggested a possible reduction in heart failure rehospitalization [3,13].

Each additional GDMT pillar was associated with incrementally more favorable remodeling and lower rehospitalization risk, supporting the concept that comprehensive neurohormonal and metabolic blockade may exert additive and potentially synergistic effects in the vulnerable post-infarction myocardium. However, as this was a retrospective observational study, residual confounding by indication cannot be excluded despite multivariable adjustment, and the observed associations should be interpreted cautiously [14,15].

### 4.1. From Therapeutic Inertia to Remodeling Biology

The underutilization of quadruple GDMT has been consistently documented across international registries [16,17,18]. However, most prior analyses have focused primarily on prescription rates and hard clinical endpoints [19]. Our findings extend this literature by linking implementation intensity directly to structural cardiac remodeling parameters, including left ventricular end-diastolic diameter (LVEDD), end-diastolic volume (LVEDV), and left ventricular mass (LVM).

The observed dose–response relationship between GDMT pillar count and reduction in LV diameter is particularly noteworthy. Ventricular dilation following AMI reflects maladaptive remodeling driven by neurohormonal activation, wall stress, inflammation, and metabolic dysregulation [20]. The four GDMT pillars target complementary pathophysiological axes: RAAS inhibition attenuates maladaptive fibrosis and afterload [21], beta-blockade reduces sympathetic overdrive and myocardial oxygen demand, and mineralocorticoid receptor antagonism mitigates fibrosis and adverse extracellular matrix remodeling [2,22], while SGLT2 inhibition modulates myocardial energetics, preload, and systemic inflammation [23,24,25,26].

The findings indicate that partial blockade may not adequately address the complex remodeling cascade, while comprehensive early intervention may reflect cumulative structural advantages. Importantly, the association remained significant after multivariable adjustment, consistent with the robustness of the dose-dependent effect. While the per-pillar LVEDD reduction of 0.120 cm is modest in absolute terms, the cumulative effect across four pillars (approximately 0.48 cm) approaches clinical significance, comparable to early remodeling effects reported in landmark monotherapy trials (e.g., SAVE, CAPRICORN). Moreover, the stronger effect was observed in the HFrEF subgroup (β = 0.204, *p* < 0.001). In parallel, numerically lower adjusted odds of heart failure rehospitalization were observed (OR 0.384), although these findings should be interpreted cautiously given the limited number of events.

### 4.2. Structural Remodeling as an Early Surrogate of Clinical Benefit

Although clinical outcome findings should be interpreted cautiously, structural reverse remodeling provides mechanistic support for the observed treatment associations. In our cohort, each additional GDMT pillar was independently associated with a greater reduction in LVEDD and substantially lower odds of HF rehospitalization. Notably, the magnitude of association with structural remodeling was more consistent than changes in LVEF [27], underscoring that volumetric and geometric indices may serve as more sensitive early indicators of therapeutic response in the post-AMI setting. This is consistent with prior evidence suggesting that LVEF recovery may lag behind volumetric reverse remodeling [13,27], partly because LVEF is load-dependent and may be influenced by acute changes in preload and afterload, rendering it a less sensitive early marker compared with direct structural measurements.

In the prespecified HFrEF subgroup—where guideline recommendations are strongest—the remodeling gradient was amplified. Patients receiving all four pillars demonstrated significant reverse remodeling, whereas those receiving fewer pillars exhibited persistent or progressive dilation. This divergence suggests that early comprehensive therapy may alter the structural trajectory of the ventricle during the critical remodeling window following infarction.

These findings provide real-world structural corroboration of the STRONG-HF paradigm [3], which demonstrated clinical benefit from rapid GDMT optimization. Our findings further support the concept that early therapeutic intensity is linked with both reduced adverse outcomes and observable morphological remodeling, which may serve as the biological foundation for enhanced prognosis.

The inclusion of patients with LVEF > 40% broadens the biological relevance of the remodeling analysis but should not be interpreted as implying equivalent guideline indication for quadruple GDMT across the entire cohort. Rather, these analyses should be viewed as exploratory, whereas the prespecified HFrEF subgroup provides the most clinically guideline-aligned estimate.

### 4.3. The Persistent Implementation Gap

Despite Class I guideline recommendations, only a minority of eligible patients—particularly those with HFrEF—were discharged on quadruple therapy [28]. The most pronounced deficit concerned SGLT2 inhibitors, echoing international data demonstrating delayed uptake of newer therapeutic classes [29,30,31]. This gap cannot be fully explained by contraindications or intolerance and likely reflects therapeutic inertia, organizational barriers, and concerns regarding in-hospital initiation. It should be noted that the study period (January 2023–October 2024) coincided with the evolving adoption of SGLT2 inhibitors following the 2023 ESC Focused Update, and a proportion of SGLT2i prescriptions may reflect continuation of pre-existing therapy (for diabetes or CKD) rather than de novo in-hospital initiation. Our retrospective data source did not reliably distinguish between these two scenarios, and the reported underutilization rate should be interpreted in this context.

Our findings highlight that underutilization is not a benign systems issue but may have direct structural and clinical consequences. The graded relationship between therapy intensity and remodeling suggests that every missed pillar represents a lost opportunity to modify disease trajectory.

These real-world quadruple therapy rates (18.8% overall, 26.2% in HFrEF) align with the persistent global guideline-to-practice gap. Contemporary US data from the Get With The Guidelines–Heart Failure registry show quadruple prescription at discharge in only 7.2–15.3% of eligible patients with new HFrEF diagnosis (2021–2023) [32,33]. In Western Europe, the Dutch TITRATE-HF registry reported ~44% quadruple use in chronic/worsening HFrEF (range 20–79% across sites) [9]. Notably, within the same Central/Eastern European region, the recent CEE-QCC Survey (2024–2025) achieved substantially higher implementation—53.5% overall and 63.9% in HFrEF—across selected Quality of Care Centers with systematic in-hospital optimization [34]. The somewhat lower rates in our single-center post-AMI cohort likely reflect the acute clinical setting, the earlier phase of SGLT2 inhibitor adoption during our study period, and routine (non-selected) cardiology practice. This comparison underscores both regional progress in CEE and the continued need for structured in-hospital initiation protocols following myocardial infarction [35].

### 4.4. Translational Implications: Beyond Prescription Rates

The conceptual contribution of this retrospective cohort study lies in reframing GDMT implementation from a binary metric of adherence to quantitative biological exposure. By modeling GDMT intensity as an ordinal variable (0–4 pillars), we suggest a continuum of benefit rather than a threshold effect. This approach aligns with emerging concepts in precision cardiovascular medicine, in which cumulative pathway inhibition produces additive gains.

From a translational perspective, these findings support structured in-hospital initiation protocols, multidisciplinary optimization pathways, and quality improvement initiatives aimed at achieving full GDMT implementation before discharge. The early post-AMI period represents a window of heightened vulnerability, but also one of substantial therapeutic plasticity; our data suggest that early intensive pharmacological intervention may help shift this window toward more favorable structural recovery.

Accordingly, the present findings should be interpreted as evidence that broader in-hospital initiation was associated with more favorable remodeling, rather than proof that target-dose optimization had been achieved. The principal strength of the study lies in the structural remodeling signal, whereas the clinical endpoint analysis remains limited by the narrow event definition and low event count.

### 4.5. Limitations

This study has several limitations inherent to its retrospective, single-center design. First, causality cannot be established, and confounding by indication remains the principal methodological concern. Patients discharged on a higher number of GDMT pillars may have been systematically healthier, had fewer contraindications, better adherence potential, or been managed by more proactive clinicians. Although multivariable adjustment included relevant clinical covariates and severity markers, unmeasured confounders—including frailty, socioeconomic status, post-discharge adherence, transient in-hospital instability, and physician judgment regarding tolerability or prognosis—may still have influenced prescribing decisions and outcomes. Notably, baseline systolic blood pressure and serum creatinine did not differ significantly between LVEF groups, suggesting that overt hemodynamic or renal differences were unlikely to be the sole drivers of treatment variation.

Second, the sample size was modest, particularly in subgroup analyses. Only 20 heart failure rehospitalization events occurred overall (17 in the HFrEF subgroup), increasing the risk of model overfitting and limiting the precision of multivariable clinical outcome estimates. Accordingly, odds ratios and confidence intervals should be interpreted cautiously and considered hypothesis-generating rather than definitive.

Third, GDMT intensity reflected the number of foundational therapy classes prescribed at discharge rather than pharmacological dose intensity. Data regarding initial dose, titration trajectory, treatment persistence, and achievement of guideline-recommended target doses were not consistently available. In addition, part of the observed treatment exposure may have represented continuation of pre-existing outpatient therapy rather than confirmed de novo in-hospital initiation.

Fourth, echocardiographic measurements were obtained during routine clinical care by different operators, introducing potential inter-observer variability. While this reflects real-world practice, it may have attenuated measured effect sizes.

Fifth, NT-proBNP values were not consistently available. As a validated marker of ventricular wall stress and prognosis, its absence limits the integration of biomarker, structural, and clinical response domains and may reduce comparability with contemporary heart failure registries and trials.

Sixth, follow-up duration was heterogeneous. Although follow-up time was incorporated into adjusted models and sensitivity analyses were performed, temporal variability may still have influenced the magnitude of observed remodeling.

Finally, clinical outcomes were primarily limited to heart failure rehospitalization. Data regarding all-cause mortality, cardiovascular mortality, broader ischemic endpoints, and more robust time-to-event analyses were not sufficiently complete for definitive assessment in this retrospective dataset.

Nevertheless, the consistency of the dose–response association across structural and clinical endpoints, together with persistence after multivariable adjustment, supports the biological plausibility of the observed relationship.

## 5. Conclusions

In this real-world post-AMI cohort, greater GDMT intensity at discharge was associated with more favorable early ventricular reverse remodeling and lower adjusted odds of heart failure rehospitalization, requiring prospective confirmation. A substantial implementation gap persisted, particularly for quadruple therapy. These findings support prospective studies evaluating rapid comprehensive GDMT initiation after AMI and strategies to improve real-world adoption.

## Figures and Tables

**Table 1:** Baseline patient characteristics stratified by LVEF.

Characteristic	All (n = 186)	LVEF ≤ 40% (n = 107)	LVEF > 40% (n = 79)	*p*-Value
Demographics				
Sex (%)	186	107 (57.52%)	79 (42.47%)	0.040
Male (%)	142 (76.35%)	84 (59.15%)	58 (40.84%)	0.020
STEMI (%)	161 (86.55%)	95 (59.00%)	66 (41.00%)	0.022
NSTEMI (%)	25 (13.44%)	12 (48.00%)	13 (52.00%)	0.84
Monovascular (%)	47 (25.26%)	32 (29.90%)	15 (18.98%)	0.013
Bivascular (%)	77 (41.39%)	38 (35.51%)	39 (49.36%)	0.90
Trivascular (%)	62 (33.33%)	37 (34.57%)	25 (31.64%)	0.12
Clinical				
Days in hospital	5.45 ± 3.5	6.05 ± 4.1	4.64 ± 2.25	0.0078
Body mass index (kg/m^2^) at index hospitalization	28.87 ± 4.87	28.37 [IQR 25.47–30.85]	29.48 [IQR 25.84–32.60]	0.13
Body mass index (kg/m^2^) at follow-up	29.04 ± 5.06	27.89 [IQR 25.33–31.09]	29.54 [IQR 26.12–32.38]	0.04
Heart rate (beats/minute)	78.41 ± 19.40	82.34 ± 17.98	73.97 ± 18.40	0.022
Systolic blood pressure (mmHg)	143.34 ± 26.39	141.15 ± 25.48	146.30 ± 27.47	0.1896
Diastolic blood pressure (mmHg)	87.13 ± 16.64	87.57 ± 17.42	86.51 ± 15.61	0.66
Comorbidities				
Smoking status (%)	112 (60.21%)	60 (56.07%)	52 (65.82%)	0.4497
Hypertension (%)	161 (86.55%)	91 (85.04%)	70 (88.60%)	0.097
Diabetes (%)	62 (33.33%)	41 (38.31%)	21 (26.58%)	0.0111
Blood sample				
LDLc (mg/dL)	114.62 ± 39.81	113.54 ± 38.45	116.08 ± 41.78	0.42
Triglycerides (mg/dL)	133.22 ± 83.29	131.26 ± 81.83	135.90 ± 85.67	0.70
Glycemia (mg/dL)	153.05 ± 68.48	151.27 ± 71.37	155.48 ± 64.72	0.36
Triglyceride/Glycemia Index	9.00 ± 0.69	8.96 ± 0.68	9.05 ± 0.71	0.37
Uric acid (mg/dL)	5.53 ± 1.70	5.62 ± 1.68	5.41 ± 1.74	0.41
Leucocytes (/mm^3^)	11.39 ± 3.38	11.28 ± 3.35	11.53 ± 3.43	0.62
Neutrophils (/mm^3^)	8.64 ± 3.32	8.46 ± 3.28	8.90 ± 3.38	0.37
Hemoglobin (g/dL)	14.41 ± 1.53	14.34 ± 1.60	14.51 ± 1.42	0.27
Troponin (pg/mL)	10,212.98 ± 14,992.02	9433.53 ± 14,342.04	11,268.69 ± 15,861.61	0.41
Creatinine (mg/dL)	1.05 ± 0.28	1.05 ± 0.29	1.05 ± 0.28	0.95
Creatinine (mg/dL) > 48 h	1.06 ± 0.30	1.03 ± 0.29	1.08 ± 0.31	0.452
Outcomes				
HF rehospitalization (%)	20 (10.8%)	17 (15.9%)	3 (3.8%)	0.0017
Days to follow-up	278.81 ± 236.80	249 [IQR 67.25–514.75]	135 [IQR 64.50–357.25]	0.80

**Table 2:** (**A**) Medication at discharge stratified by LVEF. (**B**) Other cardiovascular medication at discharge stratified by LVEF.

(**A**)				
**Medication**	**All (N = 186)**	**LVEF ≤ 40% (n = 107)**	**LVEF > 40% (n = 79)**	***p*** **-Value**
GDMT Pillars				
RAASi (%)	142 (76.3%)	80 (74.8%)	62 (78.5%)	0.53
Beta-blocker (%)	140 (75.3%)	89 (83.2%)	51 (64.6%)	0.0013
MRA (%)	160 (86.0%)	99 (92.5%)	61 (77.2%)	0.0027
SGLT2i (%)	56 (30.1%)	43 (40.2%)	13 (16.5%)	0.0001
Pillar Count				
4 Pillars (%)	35 (18.8%)	28 (26.2%)	7 (8.9%)	0.0004
3 Pillars (%)	80 (43.0%)	50 (46.7%)	30 (38.0%)	0.25
2 Pillars (%)	49 (26.3%)	22 (20.6%)	27 (34.2%)	0.047
0–1 Pillars (%)	22 (11.8%)	7 (6.5%)	15 (19.0%)	0.013
(**B**)				
**Medication**	**All (N = 186)**	**LVEF ≤ 40% (n = 107)**	**LVEF > 40% (n = 79)**	***p*** **-Value**
Loop diuretics	151 (81.1%)	94 (87.8%)	57 (72.1%)	0.0026
Thiazide diuretics	4 (2.1%)	1 (0.9%)	3 (3.8%)	
Calcium channel blockers	11 (5.9%)	5 (4.6%)	6 (7.6%)	0.76
DAPT	186	107	79	
Statins	186	107	79	
Anticoagulant	40 (21.5%)	29 (27.1%)	11 (13.9%)	0.0044

**Table 3:** Echocardiographic measurements stratified by LVEF.

Parameter	All (N = 186)	LVEF ≤ 40% (n = 107)	LVEF > 40% (n = 79)	*p*-Value
Baseline				
LVEF (%)	41.2 ± 7.6	36.0 ± 4.8	48.3 ± 4.3	<0.0001
LVEDD (cm)	4.86 ± 0.62	4.99 ± 0.62	4.69 ± 0.57	0.0009
LVEDV (mL)	117.2 ± 34.0	125.2 ± 36.9	106.2 ± 26.0	0.0001
LVM (g)	225.7 ± 64.1	237.4 ± 65.9	209.8 ± 58.3	0.0035
LVMi (g/m^2^)	113.20 ± 30.07	119.89 ± 3.09	104.13 ± 2.77	0.0003
Follow-up				
LVEF (%)	45.1 ± 8.2	41.3 ± 7.8	50.3 ± 5.4	<0.0001
LVEDD (cm)	4.94 ± 0.64	5.06 ± 0.66	4.77 ± 0.56	0.0019
LVEDV (mL)	123.4 ± 40.4	133.0 ± 45.1	110.5 ± 28.6	0.001
LVM (g)	209.7 ± 52.8	215.6 ± 52.7	201.6 ± 52.2	0.073
LVMi (g/m^2^)	105.08 ± 24.80	108.93 ± 25.25	99.88 ± 23.34	0.0136
Change (Δ)				
ΔLVEF (%)	−3.93 ± 6.5	−5.31 ± 6.9	−2.06 ± 5.5	0.0007
ΔLVEDD (cm)	−0.076 ± 0.56	−0.071 ± 0.57	−0.08 ± 0.55	0.89
ΔLVEDV (mL)	−6.27 ± 29.7	−7.72 ± 33.8	−4.31 ± 23.1	0.47
ΔLVM (g)	16.04 ± 55.8	21.80 ± 57.56	8.23 ± 52.54	0.10
ΔLVMi (g/m^2^)	8.11 ± 27.79	10.96 ± 29.12	4.24 ± 25.56	0.10

**Table 4:** Echocardiographic remodeling by GDMT intensity in HFrEF patients (*n* = 107).

Parameter	4 Pillars (*n* = 28)	<4 Pillars (*n* = 79)	*p*-Value
ΔLVEF (%)	−6.5 [−10 to −1.5]	−5.0 [−10 to 0]	0.11
ΔLVEDD (cm)	+0.21 ± 0.49	−0.17 ± 0.57	0.0021
ΔLVEDV (ml)	+2.5 [−15 to 20]	−10.0 [−25 to 9.5]	0.12
ΔLVM (g)	+37.4 ± 46.3	+16.3 ± 60.4	0.09

Δ values were calculated as the difference between baseline and follow-up measurements (baseline − follow-up). Accordingly, a negative ΔLVEF reflects functional improvement, corresponding to an increase in LVEF at follow-up, whereas a positive Δ for structural parameters (LVEDD, LVEDV, and LVM) indicates favorable reverse remodeling, corresponding to a reduction in chamber size or myocardial mass at follow-up.

**Table 5:** Multivariable linear regression analysis for ΔLVEDD in the overall cohort and HFrEF subgroup: primary and clinical severity-adjusted models.

Variable	β (All Cohort)	*p*-Value	β (All Cohort, Severity-Adjusted)	*p*-Value	β (HFrEF)	*p*-Value	β (HFrEF, Severity-Adjusted)	*p*-Value
GDMT pillars (per unit)	0.120	0.004	0.109	0.010	0.204	<0.001	0.186	0.001
Age	0.007	0.069	0.006	0.111	0.015	0.001	0.014	0.005
Sex (male)	−0.178	0.052	−0.159	0.079	−0.032	0.796	−0.062	0.618
STEMI	−0.103	0.352	−0.067	0.553	−0.203	0.200	−0.217	0.188
Diabetes	−0.061	0.449	−0.040	0.629	0.013	0.897	0.020	0.848
Multivessel disease	0.196	0.028	0.185	0.036	0.102	0.368	0.092	0.424
Baseline LVEDD	0.383	<0.001	0.408	<0.001	0.295	0.001	0.312	<0.001
Days to follow-up	−0.00020	0.210	−0.00028	0.079	−0.00040	0.055	−0.00045	0.032
Killip class	—	—	−0.016	0.803	—	—	−0.004	0.954
Systolic blood pressure	—	—	0.0033	0.024	—	—	0.0031	0.124
Heart rate	—	—	−0.0042	0.031	—	—	−0.0028	0.283
Creatinine	—	—	0.179	0.161	—	—	0.102	0.543

Abbreviations: GDMT = guideline-directed medical therapy; LVEDD = left ventricular end-diastolic diameter; HFrEF = heart failure with reduced ejection fraction. Models: Model 1: adjusted for age, sex, ACS type, diabetes, multivessel disease, baseline LVEDD, and follow-up duration; Model 2: additionally adjusted for baseline clinical severity (Killip class, systolic blood pressure, heart rate, creatinine); β coefficients represent change in ΔLVEDD (cm).

## Data Availability

The data presented in this study are available on request from the corresponding author. The data are not publicly available due to privacy restrictions.

## Supplementary Materials

The following supporting information can be downloaded at: https://www.mdpi.com/article/10.3390/biomedicines14051067/s1, Table S1: Baseline characteristics according to GDMT intensity; Table S2: Multivariable linear regression for ΔLVEDD stratified by follow-up duration (overall cohort); Table S3: Multivariable linear regression for ΔLVEDD stratified by follow-up duration (HFrEF subgroup).
