# Supplementary material for: Guideline-Directed Medical Therapy Intensity, Ventricular Remodeling, and Clinical Outcomes After Acute Myocardial Infarction: A Single-Center Real-World Retrospective Cohort Study
Source: Biomedicines. 2026 May 8;14(5):1067. doi: 10.3390/biomedicines14051067 (PMC13204536; doi:10.3390/biomedicines14051067)
Supplement: Supplementary file 1 [file biomedicines-14-01067-s001.zip › biomedicines-4242183-supplementary.pdf]

Supplementary Materials

Supplementary analyses were performed to further explore baseline treatment-selection patterns and the influence of follow-up duration on remodeling outcomes.

Table S1. Baseline characteristics according to GDMT intensity

GDMT pillars	n	Age (years)	SBP (mmHg)	Heart rate (bpm)	Creatinine (mg/dL)	Killip ≥II (%)	Baseline LVEDD (cm)
0	2	72.0	125.0	90.0	1.4	0.0	4.8
1	20	63.9	135.0	72.4	1.1	10.0	4.5
2	49	60.8	138.0	74.7	1.0	18.4	4.8
3	80	59.4	147.0	79.5	1.1	17.5	4.9
4	35	60.4	147.0	83.9	1.0	17.1	5.1

Continuous variables are presented as means; Killip ≥II is presented as percentage.

Table S2. Multivariable linear regression for ΔLVEDD stratified by follow-up duration (overall cohort)

Variable	β (<6 months)	p-value	β (≥6 months)	p-value
GDMT pillars	0.084	0.150	0.132	0.051
Age	0.003	0.585	0.008	0.192
Male	−0.260	0.030	−0.070	0.633
STEMI	0.035	0.803	−0.283	0.167
T2DM	0.053	0.635	−0.146	0.281
Multivessel disease	0.290	0.068	0.144	0.223
Baseline LVEDD	0.454	<0.001	0.374	<0.001
Killip class	−0.098	0.322	0.041	0.645
Systolic BP	0.002	0.348	0.005	0.035
Heart rate	−0.005	0.099	−0.003	0.327
Creatinine	0.199	0.221	0.100	0.640

Table S3. Multivariable linear regression for ΔLVEDD stratified by follow-up duration (HFrEF subgroup)

Variable	β (<6 months)	p-value	β (≥6 months)	p-value
GDMT pillars	0.081	0.345	0.224	0.011
Age	0.010	0.165	0.015	0.067
Male sex	−0.075	0.641	−0.110	0.589
STEMI	−0.133	0.514	−0.232	0.423
T2DM	0.117	0.449	0.050	0.760
Multivessel disease	0.591	0.041	0.020	0.898
Baseline LVEDD	0.356	0.007	0.289	0.023
Killip class	−0.027	0.814	0.039	0.693
Systolic BP	−0.001	0.696	0.006	0.055
Heart rate	−0.002	0.643	−0.003	0.371

Variable	β (<6 months)	p-value	β (≥6 months)	p-value
Creatinine	0.005	0.982	0.173	0.519

As an additional exploratory sensitivity analysis, remodeling was normalized to follow-up duration (ΔLVEDD per month). Higher GDMT intensity was associated with a numerically greater rate of reverse remodeling, although this did not reach conventional statistical significance ($\beta = -0.030$ cm/month per pillar, $p = 0.083$). These findings were directionally consistent with the primary analyses.
